# NELL1 membranous nephropathy: clinical associations provide mechanistic clues

**DOI:** 10.3389/fneph.2024.1323432

**Published:** 2024-03-26

**Authors:** Nicole K. Andeen, Vanderlene L. Kung, Rupali S. Avasare

**Affiliations:** ^1^ Department of Pathology and Laboratory Medicine, Oregon Health & Science University, Portland, OR, United States; ^2^ Department of Medicine, Division of Nephrology and Hypertension, Oregon Health & Science University, Portland, OR, United States

**Keywords:** NELL1, membranous nephropathy, lipoic acid, NSAID, glomerular disease, drug-induced kidney disease

## Abstract

Neural epidermal growth factor-like 1 (NELL1) membranous nephropathy (MN) is notable for its segmental deposit distribution, IgG1 dominant deposits, and comparatively high rate of spontaneous remission. It has been associated with a variety of exposures and secondary conditions, specifically use of thiol-containing medications – including lipoic acid, bucillamine, and tiopronin – as well as traditional indigenous medications (TIM) particularly those with high mercury content, and non-steroid anti-inflammatory drugs (NSAIDs). Malignancies, graft *vs.* host disease (GVHD), infection, and autoimmune conditions have also been associated with NELL1 MN. Herein, we provide a detailed summary of the clinicopathologic features of NELL1 and associations with underlying conditions, with a focus on treatment and outcomes. Rare cases of dual NELL1 and phospholipase A2 receptor (PLA2R) positive MN are reviewed. Genome-wide association study of *NELL1*, role of NELL1 in other physiologic and pathologic processes, and connection between NELL1 MN and malignancy with relevance of NELL1 tumor staining are examined. Finally, relationships and potential disease mechanisms of thiol- and mercury- associated NELL1 MN are discussed.

## Introduction

Membranous nephropathy (MN) is an autoimmune disease characterized by autoantibodies directed against podocyte antigens (1–3). MN is the most common cause of primary nephrotic syndrome (urinary protein loss > 3.5 grams per day with edema, hypoalbuminemia, and hyperlipidemia) in adults, with a prevalence estimated between 2-17 million cases per year (4–7). Patients with MN experience significant morbidity related to manifestations of nephrotic syndrome and chronic kidney disease. Although most patients have preserved kidney function at diagnosis, untreated disease can progress to end-stage kidney disease (ESKD) in up to a third of patients (4). The 2021 Kidney Disease Improving Global Outcomes (KDIGO) guidelines therefore advise consideration of immunosuppressive therapy in patients with moderate and high-risk disease (8).

Although falling under the same diagnostic terminology, clinical and pathologic features of “idiopathic” versus “secondary” MN have long been recognized. In 2009, Beck et al. discovered the major antigen involved in disease pathogenesis of idiopathic MN, the phospholipase A2 receptor (PLA2R) (2). Subsequently, with the use of mass spectrometry to identify proteins enriched in glomerular immune deposits, the known autoantigens in MN have rapidly expanded in the last 10 years, leading to autoantigen-based MN classification (9). The most common MN autoantigen remains PLA2R, which has been extensively phenotyped and provides a framework by which to study newly discovered antigens. Other notable MN antigens have been described and reviewed elsewhere (10), but include: thrombospondin type-1 domain containing 7A (THSD7A, associated with primary MN and malignancy) (11), neural epidermal growth factor-like 1 (NELL1, described below) (12, 13), protocadherin 7 (no definite association; subset with autoimmunity or malignancy) (14), high-temperature requirement A serine peptidase (HTRA1, no identified association) (15), Semaphorin 3B (associated with childhood MN) (16), Netrin G1 (no identified association) (17), FAT1 (associated with graft vs. host disease, GVHD) (18), contactin 1 (associated with chronic inflammatory demyelinating polyneuropathy, CIDP) (19), neuron derived neurotrophic factor (NDNF, associated with syphilis) (20), proprotein convertase subtilisin/kexin type 6 (PCSK6, associated with use of nonsteroidal anti-inflammatory drugs, NSAIDs) (21), and lupus MN associated antigens: exostosin 1/2 (22), neural cell adhesion molecule (NCAM1) (23), transforming growth factor beta receptor 3 (TGFBR3) (24) and others (25).

Given their relative rarity and more recent recognition, data for some of these antigens is based on fewer patients and is actively evolving. However, NELL1 has emerged as the second most common autoantigen in MN after PLA2R, with some distinct clinical associations and pathologic features. In this review we discuss the clinicopathologic characteristics of NELL1 MN. In addition, we summarize cases of MN expressing both PLA2R and NELL1, and we review mechanistic theories in NELL1 MN. As with other forms of MN, the factors driving development of anti-NELL1 autoantibodies are poorly understood but necessary to elucidate if we are to better treat and prognosticate patients with NELL1 MN.

## Clinicopathologic studies of NELL1 MN

In 2019, two groups identified neural epidermal growth factor-like 1 (NELL1) podocyte antigen in a subset of MN with distinct histologic and immunologic features, generally segmental glomerular capillary loop subepithelial deposits with IgG1 dominant staining (12, 13). Although initially suggested to be largely a primary MN comprising 16% of PLA2R negative MN (12), secondary associations were subsequently discovered and account for the majority of cases in some studies (up 89% in our recent series) (26). The strength of these associations, disease prevalence and epidemiologic characteristics, and clinical outcomes vary by study and location, and are summarized in **Table 1**.

**Table 1:** Series and case reports of NELL1 MN patients.

Series/reference	#	Location	% of MN biopsies	Age, sex	Clinical associations and conditions	Biopsy findings in NELL1 MN	Treatment	Outcomes
Sethi et al. KI 2020 (12)	34	US, France, Belgium	16% of PLA2R neg MN	63; 52% male	12% with malignancy, others presumed primary	Segmental in 18%, EM stage I or II in all	NA	NA
Caza et al. KI 2020 (13)	91	US	3.8% of PLA2R and THSD7A neg MN	67; 58% male	33% with malignancy, 24% diabetes 2% IBD	Incomplete in 93%, IgG1 (co)dominant in 96%, 24% mes deposits	RAAS blockade in 54%, IS in 25%	At 10 months, 61% remission (34% CR, 27% PR); 39% no remission
Wang et al. CJASN 2021 (27)	15	China	35% of PLA2R and THSD7A neg MN	49; 73% female	0% malignancy	IgG4 (co)dominant in 67%	IS in 83%	At 25 months, 92% in remission (33% CR)
Kudose et al. KI 2021 (28)	5	US	29% of segmental MN*	58; 4/5 female	1/5 malignancy	Segmental	RAAS blockade in 5/5, no IS	All in remission (3 CR, 2 PR)
Kudose et al. KIR 2021 (29)	2	US	2/9 of GVHD-associated MN	66; 2/2 male	2/2 GVHD, 0 active malignancy	Segmental, TBM deposits	NA	NA
Munch et al. AJT 2021 (30)	1	Germany	NA	56; 1/1 male	Allograft NELL1 MN, 25 years post-transplant. New ALS, started on LA and DMPS 8 weeks prior	Concurrent IgA nephropathy; no rejection	LA and DMPS cessation, RAAS blockade	At 4 months, PR
Spain et al. KI 2021 (31)	4	US	NA	60; 4/4 female	4/4 on LA 2 with multiple sclerosis, 1 celiac disease, 0 malignancy	Segmental in 4/4	LA cessation in 4/4, RAAS blockade in 4/4. No IS	At 9 months, all in remission (2 CR, 2 PR)
Caza et al. KI 2021 (32)	115	US	NA	Of 15 on LA: 68; 60% female	13% on LA (1/15 also with active malignancy). 8/15 on LA had diabetes	NELL1 MN	12/13 on LA remained on drug. IS in 4/15 on LA	At 14 months, remission in 11/15 on LA (7/15 CR, 4 PR); 27% no remission
Miller et al. Ped Neph 2022 (33)	1	US	4% adolescent non-lupus MN#	16; 1/1 female	1/1 obesity	IgG1 dominant, EM stage 1	NA	CR in 1/1
Iwakura et al. Sci Rep 2022 (34)	4	Japan	8.5% of secondary MN, 1.5% of primary MN	69; 4/4 male	2/4 with RA, 1 NSAIDs, 0 malignancy	IgG4+ in 1/4	IS in 2/4	At 6 months CR in 2/3
Dinesh et al., Glom dz 2022 (35)	1	US	2/5 HIV-associated MN	65; 1/1 male	Treated HIV with undetectable viral load	Segmental, “full house” IF	RAAS blockade	At 7 months, CR
Kurien et al. KI 2022 (36)	64	India, US	34% of all MN; 88% TIM-associated MN	TIM-MN: 41; 63% female	91% on TIM; 9/9 of TIM-MN patients had elevated blood mercury levels. ~2% malignancy, 0% autoimmune disease. In TIM-MN: 26% diabetes	NELL1 MN	TIM cessation, RAAS blockade in most. IS in 24%	For TIM-MN, at 3.5 months, remission in 74% (46% CR, 28% PR)
Tsuji et al. medRxiv 2022 (37)	16	Japan	13% of MN; 79% of RA-associated MN (56% of RA-MN on bucillamine)	75; 69% male	31% autoimmune disease including 19% RA on bucillamine, 13% malignancy, 63% diabetes	IgG1 dominant; DN in 10%; EM stage I or II in 90%	IS in 44%, RAAS in 31%	At 9 months, 77% remission
Miyazaki et al. KIR 2023 (38)	10	Japan	4.5% of MN	NA	6/10 with RA, 3 on bucillamine, 1 on adalimumab, 1 HCV	NELL1 MN	For 3 on bucillamine, all discontinued drug, IS in 1	For 3 on bucillamine with follow up, all in remission
Takahashi-Kobayashi et al. KIR 2023 (39)	8	Japan	9.1% of MN	NA	1/8 RA on bucillamine, 1 IgG4 related disease	NELL1 MN	NA	NA
Sethi CKJ 2023 (40) and Zubidat et al. KIR 2023 (41)	(review)	US	(review)	(review)	NELL1+ in 4/19 tested cases of sarcoidosis associated MN, 2/8 HBV, 1 HCV, 4 NSAIDs, 3 auto-immune disease, 1 IVIG, 1 HSCT^	NELL1 MN	NA	NA
Santoriello et al. KIR 2023 (42)	1	US	NA	53; 1/1 male	Tiopronin, no malignancy	Segmental	Tiopronin cessation	At 6 months, PR in 1/1
Zhu et al. Kid Dis 2023 (43)	58	China	43% of segmental MN*	40; 78% female	9% TB, 7% malignancy	Segmental, EM stage I or II in 93%, 7% mesangial deposits	NA	At 12 months, 71% remission (7/14 CR, 3/14 PR), 29% no remission
Sultan et al. Clin Tox 2023 (44)	3	India	NA	3/3 female same family, ages 17, 19, 39	Skin lightening cream with high mercury content; elevated blood mercury levels; hypothyroidism	NELL1 MN	IS in 2/3	Remission in 2/3
Nimkar et al. KIR 2023 (45)	1	US	NA	71; male	History of malignancy but NED; on CPI	Weak PLA2R staining	Pembrolizumab cessation	At 2 months, in remission
Avasare et al. KIR 2024 (26)	70	US	NA	66; 53% male	36% on LA, 23% autoimmune disease, 27% diabetes, 27% NSAIDs, 10% recent malignancy	Segmental in 55%; EM stage I or II in 82%; IgG1 (co)-dominant in 86%; mesangial deposits in 16% extra-glomerular deposits in 12%, DN in 8%	IS in 29%. For 25 on LA: LA cessation in 88%, IS in 4%	At 11 months, 72% remission (52% CR). For 25 on LA: 88% remission (72% CR)

ALS, amyotrophic lateral sclerosis; Bucillamine, similar chemical structure to D-penicillamine; CPI, immune checkpoint inhibitor; CR, complete remission; DMPS, dimercaptopropane sulfonate; DN, diabetic glomerulopathy; EM, electron microscopy; GVHD, graft vs host disease; HBV, Hepatitis B virus; HCV, Hepatitis C virus; HIV, human immunodeficiency virus; HSCT, hematopoietic stem cell transplant; IBD, inflammatory bowel disease; IF, immunofluorescence; IS, immunosuppression; LA, lipoic acid; MN, membranous nephropathy; NA, Not applicable or not available; NED, no evidence of disease; Neg, negative; NELL1, neural epidermal growth factor like 1; NSAID, non-steroidal anti-inflammatory; PLA2R, Phospholipase A2 receptor; PR, partial remission; RAAS, renin-angiotensin system; RA, rheumatoid arthritis; TB, tuberculosis; TBM, tubular basement membrane; THSD7A, thrombospondin type 1 domain-containing 7A; TIM, traditional indigenous medications, the most common of which was Swasa Kalpa which had very elevated mercury content. Tiopronin: thiol agent for cystinuria.

Listed age is mean or median, as provided by study. Results given in % in case series with ≥ 10 patients, and as fractions when <10 patients present.

*segmental MN was 2.5% of MN in Kudose et al. and 0.51% of adult MN in Zhu et al.

#NELL1 positive in 1/25 MN biopsies from adolescents aged 13-20.

^associations described in review article.

**Figure 1:**
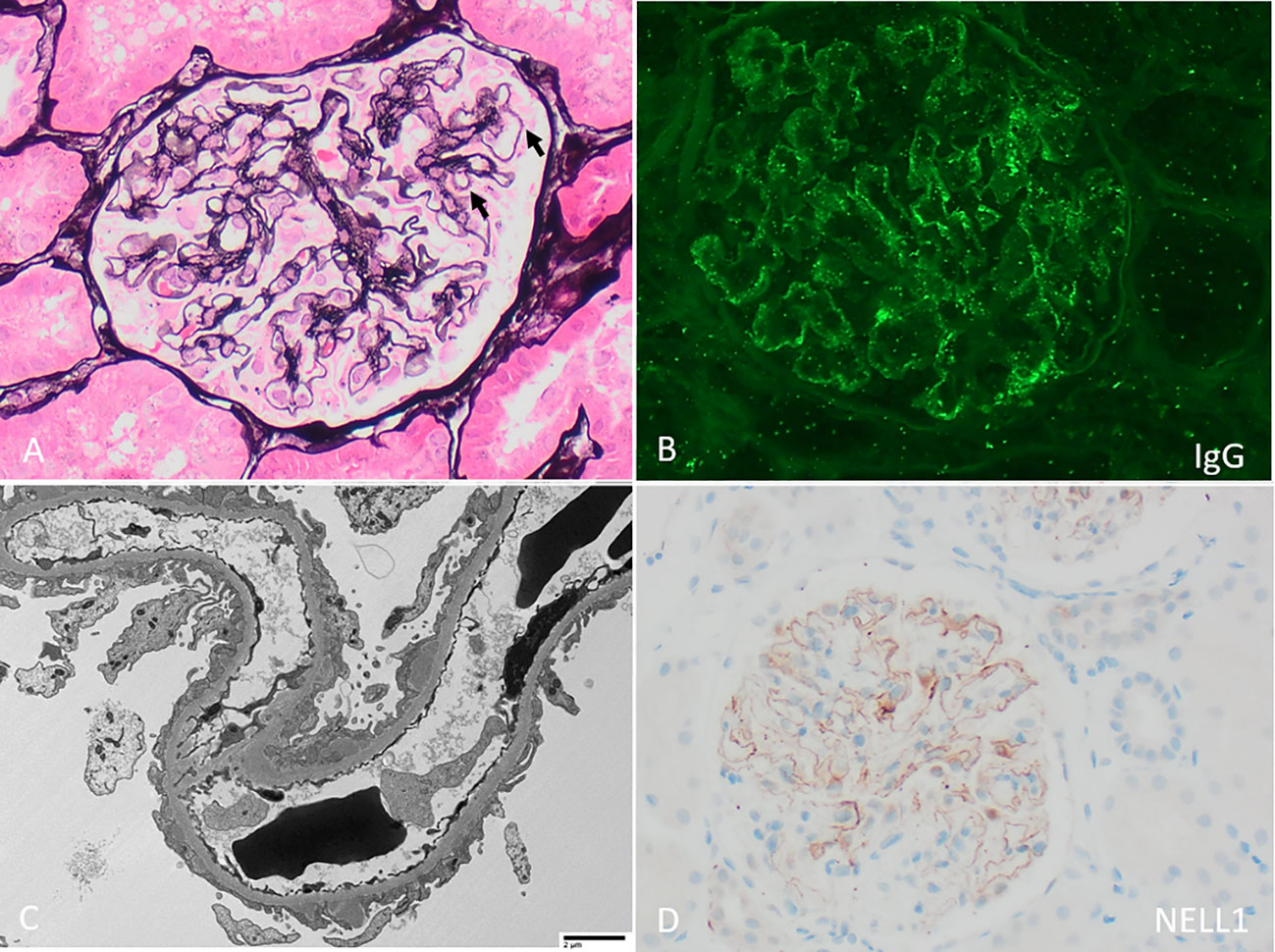
Neural epidermal growth factor-like 1 (NELL1) membranous nephropathy with **(A)** “stiff” appearing capillary loops with faint rarefactions of the glomerular basement membrane and subepithelial immune deposits (arrows, Jones stain 400x). **(B)** By immunofluorescence, there is segmentally distributed granular peripheral capillary wall staining for polyclonal IgG. **(C)** By electron microscopy, irregularly distributed subepithelial immune deposits are present, with associated podocyte foot process effacement (direct magnification 1900x). **(D)** By immunohistochemistry, there is corresponding incomplete capillary wall staining of immune deposits for NELL1.

On presentation, patients with NELL1 MN have nephrotic range proteinuria (approximately 35-80%), often preserved kidney function (in 70-84%), and are usually adults with a median age in their 60’s although adolescent cases have been reported (**Table 1**) (12, 13, 26, 43). NELL1 MN has been reported in wide spectrum of racial and ethnic backgrounds; taken together, larger studies show a relatively similar male to female distribution although this varies significantly by study and underlying association (**Table 1**). Kidney biopsies (**Figure 1**) show the characteristic MN pattern of subepithelial immune deposits but these are usually segmental to incomplete (55-94%, rather than global as with PLA2R MN), and may be subtle by light microscopy (12, 13, 26). The immune deposits are usually IgG1 (co)dominant (86-96%), and Stage I-II by electron microscopy (82-93%); mesangial (7 - 24%) and occasional subendothelial and/or extraglomerular deposits of IgG may be present (12, 13, 26, 43). The immune deposits are positive for NELL1 by immunohistochemistry, confirming the diagnosis; corresponding anti-NELL1 antibodies have also been identified in the sera of affected patients (12, 13). Although anti-PLA2R serum testing can be used in select patients for a non-invasive diagnosis and monitoring of PLA2R MN (46, 47), anti-NELL1 serum testing is not yet widely commercially available. Notably, NELL1 MN has relatively similar pathologic features regardless of underlying etiology. However, clinical outcomes are impacted by the details of – or discovery of – the underlying exposure or condition (26), as discussed below.

NELL1 MN is associated with use of thiol-containing medications, namely lipoic acid (31, 32), bucillamine (38), and tiopronin (42), and it is plausible that NELL1 was the causative auto-antigen for many cases historically associated with thiol compounds. Traditional indigenous medications (TIM) (36) and skin products (44) are associated with NELL1 MN, and tested medications and compounds have had high mercury content with corresponding elevated blood mercury levels in affected individuals (36). The largest series of TIM-associated MN was reported from India, where NELL1 MN comprised 34% of all MN and 88% of TIM-MN (36), again suggesting that auto-reactivity to the NELL1 antigen specifically may account for most historically reported mercury-associated MN. The remission rate of NELL1 MN in patients on TIM (74% at 3.5 months) (36) or lipoic acid (88% at 11 months, without immunosuppression) (26) is quite high, and significantly higher than other NELL1 MN associations (26), likely related to relative simplicity of discontinuing the exposure (i.e. compared with treating a complex underlying condition). Thus there is a potentially large subset of patients with NELL1 MN secondary to thiol-containing medications or heavy metal exposures who have an excellent prognosis and likely do not require immunosuppression.

Another major underlying condition is malignancy, reported in in 0-33% of patients depending on study (13, 27, 36), substantially higher than that seen with PLA2R MN (~4%) (4, 13, 48). Of reported patients, 9 of 12 (75%) with oncologic remission also had remission of proteinuria (13, 42), supporting this link. Other conditions include infections – such as human immune deficiency virus (HIV) (35), hepatitis B and C (HBV, HCV) (40), tuberculosis (43). MN in the setting of hematopoietic stem cell transplant and graft-versus-host disease (GVHD) can occur with the NELL1 antigen (29) although is much more commonly associated with the protocadherin FAT1 antigen (18). Autoimmune disease, seen in 0-31% of reported patients (13, 26, 36, 37), including sarcoidosis (40, 41), rheumatoid arthritis (especially patients treated with bucillamine) (37–39) and others may also be associated with NELL1 MN. Finally, NSAIDs have also been associated with NELL1 MN (26, 34, 40) (up to 27%), though are also associated with the PCSK 6 antigen (21) and clinical correlations between NSAID cessation and remission are currently less well-documented than for thiol- or mercury- associated NELL1 MN.

Notably, series of NELL1 MN from India (36) and China (27) generally lack the associations with autoimmune disease or malignancy reported from the US, Europe, and Japan, and additional underlying genetic and/or environmental factors may contribute to disease development in different groups. Higher rates of remission tend to be seen in association with thiol or mercury exposures, and potentially with oncologic remission (13).

## Dual PLA2R and NELL1 positive MN

PLA2R and NELL1 are the most commonly implicated antigens in MN and have distinct clinical associations, but rare cases have been reported as dual positive (summarized in **Table 2**). Of seven cases, two describe patients with a low-titer (27) or borderline (50) serum anti-PLA2R antibodies at time of biopsy, one with positive tissue PLA2R (27), one with negative tissue PLA2R (50). The first went into remission but relapsed with high-titer anti-PLA2R antibodies (without anti-NELL1 antibodies) (27), and the second progressed to have a positive serum anti-PLA2R antibody (with persistent anti-NELL1 antibodies) (50). A third patient had an initial biopsy with PLA2R+ NELL1- MN (no serum available), treated conservatively, with relapse >10 years later and biopsy demonstrating dual PLA2R+ NELL1+ MN with corresponding serum antibodies to both PLA2R and NELL1 (37). In two cases, PLA2R glomerular staining is described as weak with an incomplete capillary loop distribution and was associated with a negative serum anti-PLA2R antibodies at the time of biopsy (35, 45, 49), which may represent false-positive tissue reactivity.

**Table 2:** Cases of dual PLA2R and NELL1 positive MN.

Case/reference	#	Clinical characteristics	Biopsy findings	Serologic findings	Follow up
Sethi et al. JASN 2021 (3)	1	Unpublished, described in review article	Dual PLA2R+ and NELL1+ based on mass spectrometry	NA	NA
Wang et al. CJASN 2021 (27)	1	68 year old Chinese female	Dual PLA2R+ and NELL1+ deposits, IgG1 and IgG4 codominant	Low titer anti-PLA2R pos; anti-NELL1 neg	Treated with cyclosporin, with remission and neg anti-PLA2R. Relapse with high titer anti-PLA2R Ab. Serum anti-NELL1 remained negative
Dinesh et al. Glom dz 2022 (35) & Charu et al. BMC Neph 2020 (49)	1	Treated HIV with undetectable viral load	Dual PLA2R+ (weak, incomplete) and NELL1+ deposits. Global subepi, no mes or subendo deposits. IgG4 dominant.	Anti-PLA2R neg; anti-NELL1 not tested	NA
Tsuji et al. medRxiv 2022 (37)	1	Male in 60s, type 2 diabetes	Initial biopsy PLA2R+, NELL1- Second biopsy >10 years later: dual PLA2R+, NELL1+	Not available at first biopsy. At second biopsy: low titer anti-PLA2R pos, and anti-NELL1 pos	After initial biopsy: conservative therapy and remission. Relapse >10 years later: second biopsy showed dual PLA2R+ and NELL1+
Inoue et al. Kid Med 2023 (50)	1	Japanese male in 70s with HTN. No diabetes or malignancy	NELL1+, PLA2R-. IgG1 dominant, subepi deposits	Borderline anti-PLA2R; anti-NELL1 pos	Conservative therapy; worsened 5 months later, developed pos anti-PLA2R with persistent anti-NELL1. Treated with IS with remission and improvement in both Abs
Nimkar et al. KIR 2023 (45)	1	71 year old male, lung cancer with NED, treated with CPI	NELL1+ PLA2R+ (weak). Subepi, no mes or subendo deposits	Anti-PLA2R neg; anti-NELL1 not tested	Cessation of pembrolizumab, remission at 2 months
Avasare et al. KIR 2024 (26)	1	NA	Dual PLA2R+ and NELL1+. Global subepi, no mes or subendo deposits	NA	NA

Ab, antibody; CPI, checkpoint inhibitor therapy; Mes, mesangial; NA, not available; NED, no evidence of disease; Neg, negative; NELL1, neural epidermal growth factor like; PLA2R, Phospholipase A2 receptor; Pos, positive; Subendo, subendothelial; Subepi, subepithelial.

Taken together, limited data from these case reports suggest that MN with weak incomplete tissue PLA2R and negative serum anti-PLA2R antibodies results may benefit from additional MN antigen testing including NELL1. Patients with borderline or low-titer anti-PLA2R serum antibodies may experience a rise in anti-PLA2R titer or disease recurrence, even if the biopsy shows MN with NELL1+ and PLA2R- deposits. Finally, dual PLA2R and NELL1 tissue staining with corresponding autoantibodies may rarely exist or evolve over time. Although epitope spreading is well-described within the PLA2R molecule (51–54), mechanisms for relationships with other MN antigens are unknown.

## NELL1 in physiology and disease

The *NELL1* gene is located on chromosome 11 and encodes an 810 amino acid protein with a secretory signal peptide, N-terminal thrombospondin-1-like molecule (also called laminin G domain), five von Willebrand factor-like repeats with associated cysteine residues, and six epidermal growth factor (EGF)-like repeats (55–57). It is glycosylated, secreted as a 400 kDa homotrimer, and acts as cell-signaling molecule which binds to and is phosphorylated by protein kinase C-β1 (PKC- β1) (56). In human bulk tissue RNA-seq studies, *NELL1* transcripts are enriched in brain, kidney, prostate, and testis (55). *NELL1* also appears conserved across species, with 95% nucleotide homology between mice and humans (58–60). The function(s) of NELL1 in the kidney are not known. A single cell RNA-seq study of healthy human kidney, identified *NELL1* transcripts in loop of Henle and distal tubular cells; in the glomerulus, it appears to be expressed by podocytes without significant expression by mesangial or endothelial cells (61–63) Future studies examining if and how autoimmunity and injury affect NELL1 expression within the kidney will be informative.

With regard to other human diseases, *NELL1* is best studied for its role in osteoblast differentiation. Overexpression of *NELL1* was identified within the prematurely fused coronal sutures in patients with non-familial, non-syndromic craniosynostosis (58, 64). Transgenic mice overexpressing the *Nell1* gene recapitulate this phenotype, with overgrowth of skull bones and premature suture closure (58). In oral health, it may also play a role in angiogenesis in human dental pulp stem cells (65), contribute to bone regeneration in periodontitis (66), and promote progression in osteosarcoma (67).

Conversely, downregulation of *Nell1* inhibits osteoblast differentiation (58). Mice with *Nell1* point mutations and severe loss of expression have skeletal defects in cranial vault, vertebrae, and ribs (56), supporting the protein’s role in intramembranous and endochondral ossification. In these mice, loss of *Nell1* function leads to reduced expression of genes encoding extracellular matrix proteins, the most severe of which include collagen 5 alpha 3 subunit (*Col5a3*), tenascin (*Tnxb*), proteoglycan 4 (*Prg4*), thrombospondin 3 (*Thbs3*) (56). Chondrocyte-specific *Nell1* inactivation impedes growth and mineralization of the appendicular skeleton (68), highlighting its importance in endochondral ossification outside the skull. Notably, kidney disease is not described in mouse models of overexpression or loss of expression of *Nell1*, although these studies were predominantly focused on the musculoskeletal system (56, 58).

In human genetic studies, a review (60) of genome wide association studies (GWAS) highlighted *NELL1* single nucleotide polymorphisms (SNPs) of genome wide significance (P < 5 x 10^-8^) or suggestive significance (P < 5 x 10^-5^) in triglyceride metabolism, autism, multiple sclerosis, inflammatory bowel disease (IBD), chronic periodontitis, non-small cell lung cancer (60), as well as in osteoporosis (68, 69). *NELL1* deletion has also been described in a 3-year-old with short stature, macrocephaly, and delayed fontanelle closure (70). Overall, a specific association with kidney disease in patients with *NELL1* SNPs has not been identified. However, these genetic studies corroborate the discussed roles of NELL1 in bone and dental health, and raise questions about potential genetic predispositions in the small subset of patients with NELL1 MN in the setting of multiple sclerosis (31) or IBD (13).

## Mechanistic theories in NELL1 MN

The major clinical associations in NELL1 MN point to potentially differing underlying mechanisms, specifically those linked to exposure to thiol-containing medications or mercury, and malignancy. There is less data regarding potential mechanisms for NELL1 MN associated with autoimmune disease, GVHD, NSAIDs, and idiopathic NELL1 MN, and these will not be further discussed here.

The reduced form of lipoic acid (dihydrolipoic acid) contains two thiol, or sulfhydryl groups, each of which consists of a sulfur atom bonded to hydrogen and an alkyl group (R-S-H). Structurally similar thiol groups are present in D-penicillamine, captopril, bucillamine, and tiopronin (42). Of the thiol-containing medications associated with NELL1 MN, lipoic acid is potentially unique in that it is an antioxidant naturally synthesized by various plant and mammal species, including humans, which functions in the mitochondria as an enzyme cofactor (71). Lipoic acid is chiral and natural forms exist as the R enantiomer, whereas ex vivo synthesized lipoic acid supplements contain a racemic mixture of both R and S enantiomers (71–73), which have potentially differing biologic effects. Neither specific dose toxicity nor specific manufacturers have been connected to lipoic acid associated NELL1 MN (26, 31). Adverse immune events associated with thiol compounds also include pemphigus, rash, oral mucosal ulcers, and, for lipoic acid, insulin autoimmune syndrome in genetically susceptible individuals (HLA-DRB1*0406 and DRB1*0403) (42, 74–77). Mechanisms for thiol-induced MN are likely related to thiol-disulfide exchange, altering tertiary and quaternary protein structure and potentially generating neo-epitopes (42). Anti-PLA2R auto-antibodies also bind primarily to a cysteine-rich domain of PLA2R containing disulfide bones (78), and this and other auto-antibodies in MN have been identified specifically under non-reducing conditions (2, 4) (except for Semaphorin 3B), suggesting the importance of these disulfide bonds in MN autoantigen development outside the setting of thiol exposure.

Mercury-associated NELL1 MN may have a related mechanism, as mercury shows high-affinity binding to sulfhydryl groups (79). A variety of conditions, autoimmunity, and autoantibodies have been linked to mercury (80) (associations and mechanisms recently reviewed elsewhere (79)). Autoantibody development to laminins in particular is a common finding in experimental mercury exposure (80) and is particularly relevant given the role of these extracellular matrix glycoproteins in the glomerular basement membrane (81) and the presence of a laminin G domain in the NELL1 protein (57). However, laminins are a large family of proteins, and in one study of workers with chronic mercury exposure, no significant differences were found among mercury workers with anti-laminin antibodies (n=8) vs. mercury exposed without anti-laminin antibodies (n=54) vs. controls (n=60) for kidney parameters (including proteinuria, albuminuria, creatinine, etc.), nor was there a dose relationship between mercury exposure (in blood and urine) and development of anti-laminin antibodies (82). Although small and with older techniques, this study provides some evidence against a direct mechanistic relationship between anti-laminin antibodies and mercury-associated MN, and suggests that mercury-associated autoimmunity in humans is not dose-dependent and is impacted by other, modifying factors (82).

Similar to THSD7A (83, 84), the association between NELL1 MN and malignancy is made further intriguing by the presence of tumor staining in reported cases, usually of solid tumors like breast (13, 85) or prostate cancer for NELL1. However, these initial reports often lack examination of a control group (i.e., NELL1 immunostaining in tumors from patients without NELL1 MN). NELL1 immunoreactivity has been documented in wide variety of carcinomas, including but not limited to the colon, breast, prostate, lung, liver, as well as in melanoma (86). Larger studies focusing on neoplasms identified THSD7A expression in a wide variety of neoplasms (87), raising questions about how well immunohistochemically-detected tumor antigen expression will associate with development of MN in an individual patient. Future studies of NELL1 immunoreactivity in tumors may be useful in this regard. Additionally, demonstration of an association between NELL1 MN activity and/or anti-NELL1 antibodies with tumor regression would further support the association.

## Discussion and future directions

In addition to defining the presenting clinical and pathological associations, outcome studies in NELL1 MN have revealed that remission may be largely influenced by underlying association, highlighting the importance of an etiology-based as well as antigen-based classification of MN. In the case of certain thiol- or mercury- containing medications, the relative ease of identifying and removing the exposure may account, at least in part, for the high remission rate. This attractive theory does not explain all cases, however, such as patients that continue or restart the same medication without persistence or recurrence of NELL1 MN. Nor does it account for the relatively low burden of detected disease despite the broad use of many of these supplements which, in the case of lipoic acid, has a generally innocuous safety profile in large studies (31, 88). It is likely that genetic factors play a role in disease development, and future GWAS studies accounting for the geographic and/or ethnic heterogeneity of NELL1 MN may further elucidate genetic susceptibility in patients with exposure-related NELL1 MN.

The driving mechanisms in NELL1 likely vary according to associated condition, and differences in remission rates may also reflect complexity or reversibility of these biologic mechanisms (i.e. in addition to simplicity of removing an exposure). Future studies correlating anti-NELL1 serum titers to underlying association, disease activity, and details of epitope antigenicity – similar to studies for PLA2R with the added focus of underlying association in some patients – may inform how these clinically-identified etiologies shape the immunologic aspects of disease.

Though the mechanistic underpinnings of NELL1 MN are not fully elucidated, observational findings on clinical associations have already had a significant impact on patient care. In our practice, those diagnosed with NELL1 MN undergo a thorough review of medication and supplement use, and malignancy screening. Because of the high remission rate reported in the literature, those with otherwise low risk disease are managed conservatively with non-immunosuppressive anti-proteinuric therapy for a period of 3-6 months. If the disease persists despite addressing possible secondary associations, then immunosuppressive strategies are discussed. We anticipate the development of serum anti-NELL1 antibodies will further guide the decision to escalate therapy, assuming anti-NELL1 antibodies have similar diagnostic and prognostic characteristics to anti-PLA2R antibodies. Furthermore, as mechanistic understanding increases, we anticipate the rise of specific therapies targeted to MN-antigen types with the overarching hope that patients may one day have more specific and less toxic therapies.

## Author contributions

NA: Writing – original draft, Writing – review & editing. VK: Writing – review & editing. RA: Writing – review & editing.
